# Targeted Next-Generation Sequencing in Diagnostic Approach to Monogenic Cholestatic Liver Disorders—Single-Center Experience

**DOI:** 10.3389/fped.2020.00414

**Published:** 2020-07-24

**Authors:** Patryk Lipiński, Elżbieta Ciara, Dorota Jurkiewicz, Agnieszka Pollak, Maria Wypchło, Rafał Płoski, Joanna Cielecka-Kuszyk, Piotr Socha, Joanna Pawłowska, Irena Jankowska

**Affiliations:** ^1^Department of Pediatrics, Nutrition and Metabolic Diseases, The Children's Memorial Health Institute, Warsaw, Poland; ^2^Department of Gastroenterology, Hepatology, Feeding Disorders and Pediatrics, The Children's Memorial Health Institute, Warsaw, Poland; ^3^Department of Medical Genetics, The Children's Memorial Health Institute, Warsaw, Poland; ^4^Department of Medical Genetics, Medical University of Warsaw, Warsaw, Poland; ^5^Postgraduate School of Molecular Medicine, Medical University of Warsaw, Warsaw, Poland; ^6^Department of Pathology, The Children's Memorial Health Institute, Warsaw, Poland

**Keywords:** next-generation sequencing, cholestasis, progressive familial intrahepatic cholestasis, liver transplantation, children

## Abstract

**Objective:** To evaluate the clinical utility of panel-based NGS in the diagnostic approach of monogenic cholestatic liver diseases.

**Study design:** Patients with diagnosis of chronic cholestatic liver disease of an unknown etiology underwent NGS of targeted genes panel. Group 1 included five patients (prospectively recruited) hospitalized from January to December 2017 while group 2 included seventeen patients (retrospectively recruited) hospitalized from 2010 to 2017 presenting with low-GGT PFIC phenotype (group 2a, 11 patients) or indeterminant cholestatic liver cirrhosis (group 2b, 6 patients).

**Results:** Among 22 patients enrolled into the study, 21 various pathogenic variants (including 11 novel) in 5 different genes (including *ABCB11, ABCB4, TJP2, DGUOK, CYP27A1*) were identified. The molecular confirmation was obtained in 15 out of 22 patients (68%). In group 1, two out of five patients presented with low-GGT cholestasis, and were diagnosed with BSEP deficiency. Out of three patients presenting with high-GGT cholestasis, one patient was diagnosed with PFIC-3, and the remaining two were not molecularly diagnosed. In group 2a, seven out of eleven patients, were diagnosed with BSEP deficiency and two with TJP-2 deficiency. In group 2b, three out of six patients were molecularly diagnosed; one with PFIC-3, one with CYP27A1 deficiency, and one with DGUOK deficiency.

**Conclusions:** Panel-based NGS appears to be a very useful tool in diagnosis of monogenic cholestatic liver disorders in cases when extrahepatic causes have been primarily excluded. NGS presented the highest diagnosis rate to identify the molecular background of cholestatic liver diseases presenting with a low-GGT PFIC phenotype.

## Background

Next-generation sequencing (NGS) technology including targeted gene panels, whole-exome sequencing (WES), or even whole-genome sequencing (WGS), have now opened promising possibilities to identify the molecular background of rare diseases, also in the field of pediatric hepatology and liver transplantation (1–3). It is estimated that nearly 20% of liver transplantation (LTx) procedures in children are performed due to monogenic, mostly cholestatic, liver disorders (4, 5). The identificable causes of cholestasis have grown recently due to application of NGS (5, 6). The latest guidelines from the European Society for Pediatric Gastroenterology, Hepatology, and Nutrition (ESPGHAN) have also recommended the use of modern, broad-based NGS sequencing in the proper clinical context (7).

The aim of this study was to evaluate the clinical utility of panel-based NGS in the diagnostic approach of monogenic cholestatic liver diseases.

## Materials and Methods

Five patients which were hospitalized in our center from January to December 2017 with diagnosis of chronic cholestatic liver disease of an unknown etiology were prospectively recruited to the study (group 1). Cholestasis was defined based on serum direct bilirubin concentration >1.0 mg/dL according to the latest ESPGHAN recommendations (7). The diagnosis of cholestatic liver disease of unknown etiology was established based on the following criteria:

(1) exclusion of known infectious (based on serology (IgM and IgG) of HAV, HBV, HCV, EBV, CMV, HSV-1, HSV-2, TORCH infections; bacterial cultures of blood and urine) causes of cholestasis; (2) exclusion of known metabolic (based on urine organic analysis with GC/MS profiling, acylcarnitine profile by tandem mass spectrometry analysis (MS/MS) of DBS, serum transferrin IEF, blood ammonia and lactate, serum amino acids, urinary glycosaminoglycans testing, serum very long chain fatty acids levels, urine and serum arabitol levels, morning cortisol levels, thyreotropine and free thyroxine levels, serum α1-antitrypsin levels, serum immunoreactive trypsinogen level as a part of newborn screening program) causes of cholestasis; (3) exclusion of Alagille syndrome according to the classical definition of the presence of three of five major clinical criteria (8); (4) in patients presenting with hypo-/acholic stools and/or elevated GGT cholestasis exclusion of biliary atresia (by histopathologic examination of liver biopsy specimens and/or an intraoperative cholangiography in selected cases) and other causes of extrahepatic cholestasis (by an ultrasound examination and magnetic resonance cholangiopancreatography in selected cases). The reference ranges for GGT in the 1st year of life were in accordance with Cabrera-Abreu and Green (9). In some patients presenting with intrahepatic cholestasis of an unknown etiology, the liver biopsy has been done.

The second group of patients was recruited retrospectively and included 17 patients which hospitalized in our center from 2010 to 2017 with a diagnosis of chronic cholestatic liver disease of unknown etiology. Based on clinical, biochemical, and histological presentation, this group was divided into patients presenting with low-GGT PFIC phenotype (group 2a, 11 patients) or indeterminant cholestatic liver cirrhosis (group 2b, 6 patients). The low-GGT PFIC phenotype was diagnosed based on (1) low (normal) serum GGT activity; (2) early (neonatal/infantile) onset of cholestasis; (3) exclusion of known infectious and metabolic causes of cholestasis (as mentioned above); (4) a progressive disease course; (5) liver histopathology examination resembling those in PFIC patients. The diagnosis of liver cirrhosis was based on the histopathological examination of liver biopsy specimens.

Genomic DNA was extracted by automated method (MagnaPure; Roche, CA) from peripheral blood samples of the patients. NGS of targeted genes panel, created by The Children's Memorial Health Institute's for the simultaneous sequencing of 1000 clinically relevant genes including 53 items related to cholestatic liver disorders was used as a genetic target in all patients. Coding regions (excluding non-transcribed exons) and bordering intron sequences of the following genes: *ABCB11, ABCB4, AGL, ALDOB, ATP7B, ATP8B1, BCS1L, CFTR, CLDN1, CYP27A1, DGUOK, DHCR7, EPCAM, FAH, FH, GFM1, HNF1A, HNF1B, HSD17B4, IFT172, INSR, JAG1, LIPA, MPV17, MYO5B, NEUROG3, NOTCH2* (excluding exons 1, 2, and 4 in *NOTCH2* due to highly homologous regions), *NPC1, NPC2, PEX1, PEX10, PEX11B, PEX12, PEX13, PEX14, PEX16, PEX19, PEX2, PEX26, PEX3, PEX5, PEX6, PEX7, PKHD1, POLG, SERPINA1, SLC25A13, SMPD1, TJP2, TRMU, UGT1A1, VIPAS39, VPS33B* were analyzed.

NGS was performed on a HiSeq 1500 platform (Illumina, CA) using custom NimbleGen SeqCap EZ Choice Library (Roche, CA) according to a published protocol (10). Generated reads were first merged and low-quality reads removed. Then the reads were aligned to the hg19 (GRCh37) reference human genome. Potential PCR duplicates and reads mapping to multiple genomic locations were removed. In variant calling, any call with the ratio smaller than 0.2 was assumed to be homozygous, and the rest—heterozygous. Alignments were viewed with Integrative Genomics Viewer v. 2.3.82. The detected variants were annotated using Annovar and converted to MS Access format for final manual analysis.

All the non-coding and common variants (minor allele frequency above 0.01 in the general population) were discarded. The rare variants affecting the coding regions were filtered basing on the autosomal recessive mode of inheritance and predicted consequences at the transcript level. The variants were prioritized according to the population frequency and the predicted effect on the protein.

In order to detect potentially causative variants from NGS data, we applied a computational algorithm termed Phenotypic Interpretation of eXomes (PhenIX) (11). The software evaluates and ranks detected gene variants based on pathogenicity and semantic similarity of patients' phenotype described by Human Phenotype Ontology (HPO) terms to those of known Mendelian diseases.

The nomenclature of molecular variants follows the Human Genome Variation Society guidelines (HGVS, http://varnomen.hgvs.org/) using human cDNA sequence of appropriate genes followed the Human Gene Mutation Database (HGMD, http://www.hgmd.cf.ac.uk).

The chart review of patients' medical records concerning the age of onset, as well as biochemical, histological, and molecular data were collected.

An informed and written consent was obtained from patients and their parents. Ethical approval of the study protocol was obtained from the Children's Memorial Health Institute Bioethical Committee, Warsaw, Poland.

## Results

Twenty-two patients, including 14 males and 8 females, were enrolled into the study. All patients were of Caucasian ancestry and had unrelated parents. Group 1 included 5 patients (23%) with a mean age 4 years of first signs and symptoms (median 2 months, age range 1 month−14 years) and 7 years at molecular analysis. Group 2a included 11 patients (50%) with a mean age 9 months of first signs and symptoms (median 6 months, age range 1–11 months) and 8 years at molecular analysis. Group 2b included 6 patients (27%) with a mean age 10 months of first signs and symptoms (median 2 months, age range 1 month−4 years) and 9 years at molecular analysis (in one patient *post mortem*).

Among 22 patients enrolled into the study, 21 various pathogenic variants (including 11 novel) in five different genes (including *ABCB11, ABCB4, TJP2, DGUOK*, and *CYP27A1*) were identified. The molecular confirmation of monogenic cholestatic liver disease was obtained in 15 out of 22 patients (68%). In the second study group, the positive molecular detection rate was higher than in the first group (70 vs. 60%).

In group 1, two out of five patients had presented with low-GGT cholestasis, and they were subsequently diagnosed with bile salt export pump (BSEP) deficiency. Out of three patients presenting with high-GGT cholestasis, one patient was diagnosed with progressive familial intrahepatic cholestasis type 3 (PFIC-3), and the remaining two patients were not molecularly diagnosed in NGS.

In group 2, all but one had presented with low-GGT cholestasis. Low-GGT PFIC phenotype was observed in 11 out of 17 patients while indeterminant cholestatic liver cirrhosis was reported in the remaining six patients. In patients presenting with low-GGT PFIC phenotype, the partial external biliary diversion (PEBD) procedure was performed in four of them, at a median age of 2 years (age range 0.5–6 years), due to an ineffective ursodeoxycholic acid (UDCA) therapy. In one of them, the ileal-bypass procedure had to be performed (one month after PEBD). Overall, out of all patients presenting with low-GGT PFIC phenotype, seven were diagnosed with bile salt export pump (BSEP) deficiency and two patients were diagnosed with tight junction protein 2 (TJP-2) deficiency. Two patients (including one with high-GGT cholestasis) were not molecularly diagnosed in panel-based NGS. Out of six patients presenting with indeterminant cholestatic liver cirrhosis, the living donor LTx was performed in all of them at a median age of 3 years and 4 months. Overall, in this group, one patient was diagnosed with progressive familial intrahepatic cholestasis type 3 (PFIC-3), one patient was diagnosed with bile acid synthesis defect (CYP27A1 deficiency), and one patient was diagnosed with mitochondrial depletion syndrome (DGUOK deficiency). Three patients were not molecularly diagnosed in panel-based NGS. The child diagnosed with CYP27A1 deficiency had succumbed at 3.5 years of age due to the graft failure observed 3 months after LTx.

Detailed characteristics of the clinical, biochemical, histological, and molecular phenotypes of the presented patients is summarized in Table 1.

**Table 1:** Clinical, biochemical, histological, and molecular characteristics of the studied patients.

**Patient**	**Sex**	**Age of onset/at referral**	**Results of biochemical analyses at referral**	**Results of liver biopsy (age)**	**Diagnosis**	**Surgical interventions (if done)**	**Affected gene^1^**	**Molecular variants^1^**	**Status of molecular variant + PMID (for known variants)**
**GROUP 1**									
1	M	14/15 y	ALT 114 AST 59 GGT 155 SBA 415 INR 1.05 TSB/DSB 0.6/0.4	Mild cholestasis (15 y)	PFIC		*ABCB4*	c.959C>T, p.(Ser320Phe)/c.1119+1G>A, p.?	Known (11313316)/novel
2	M	5/6 y	ALT 23 AST 44 GGT 11 SBA 430 INR 1.01 TSB/DSB 7.9/7.1	Severe cholestasis (6 y)	PFIC		*ABCB11*	c.1487A>T, p.(Asp496Val)/c.1621A>C, p.(Ile541Leu)	Known (28733223)/known (16868810)
3	F	1/1 mo	ALT 205 AST 279 GGT 58 SBA 93 INR 0.96 TSB/DSB 4.6/4.1	n.a.	PFIC no pruritus		*ABCB11*	c.1445A>G, p.(Asp482Gly)^2^^*^	Known (9806540)
4	M	2/2 mo	ALT 178 AST 286 GGT 480 SBA n.a. INR 1.32 TSB/DSB 11.2/9.2	Severe cholestasis, fibrosis stage 2 according to Batts and Ludwig classification (2 mo)	Neonatal cholestasis		No pathogenic molecular variants identified in NGS targeted gene panel		
5	F	1/1 mo	ALT 97 AST 174 GGT 200 SBA 415 INR 1.4 TSB/DSB 5.4/4.1	Severe cholestasis, no fibrosis (1 mo)	Neonatal cholestasis		No pathogenic molecular variants identified in NGS targeted gene panel		
**GROUP 2A**									
1	F	6/8 mo	ALT 60 AST 82 GGT 15 SBA 150 INR 0.95 TSB/DSB 2.5/1.9	Mild cholestasis, fibrosis stage 1 according to Batts and Ludwig classification (8 mo)	PFIC		*ABCB11*	c.1445A>G, p.(Asp482Gly)^*^/c.1558A>T, p.(Arg520^*^)	Known (9806540)/known (18395098)
2	F	9/11 mo	ALT 75 AST 91 GGT 18 SBA 144 INR 0.9 TSB/DSB 2.91/2.68	Mild cholestasis, fibrosis stage 1 according to Batts and Ludwig classification (11 mo)	PFIC		*ABCB11*	c.922C>T, p.(Leu308Phe)/c.1445A>G, p.(Asp482Gly)^*^/c.3605T>C, p.(Met1202Thr)	Novel/known (9806540)/novel
3	F	1/5 mo	ALT 145 AST 204 GGT 35 SBA 205 INR 0.9 TSB/DSB 6.2/5.2	Severe cholestasis, fibrosis stage 3 according to Batts and Ludwig classification (5 mo)	PFIC	PEBD (10 mo)	*ABCB11*	c.150+3A>G, p.?/c.150+3A>G, p.?	Novel
4	F	3/3 mo	ALT 414 AST 361 GGT 45 SBA 403 INR 0.81 TSB/DSB 8.0/6.8	Severe cholestasis, fibrosis stage 2/3 according to Batts and Ludwig classification, Hepatitis gigantocellularis (3 mo)	PFIC	PEBD → IB (6 mo)	*ABCB11*	c.1421C>T, p.(Pro474Leu)/c.1421C>T, p.(Pro474Leu)	Novel
5	F	9/9 mo	ALT 58 AST 75 GGT 20 SBA 255 INR 0.94 TSB/DSB 0.6/0.5	n.a.	PFIC		*TJP2*	c.205G>A, p.(Val69Met)/c.1099C>T, p.(Arg367^*^)	Novel/novel
6	M	6/8 mo	ALT 85 AST 100 GGT 34 SBA 200 INR 0.9 TSB/DSB 4.0/3.2	Moderate cholestasis, fibrosis stage 2 according to Batts and Ludwig classification (8 mo)	PFIC	PEBD (3 y)	*ABCB11*	c.1445A>G, p.(Asp482Gly)^*^/c.1445A>G, p.(Asp482Gly)^*^	Known (9806540)
7	M	1/5 mo	ALT 700 AST 690 GGT 89 SBA 225 INR 1.05 TSB/DSB 7.0/1.5	Severe cholestasis, fibrosis stage 1/2 according to Batts and Ludwig classification, Hepatitis gigantocellularis (5 mo)	PFIC	PEBD (5 mo)	*TJP2*	c.2037dup, p.(Ala679_Gly680fs)/c.2037dup, p.(Ala679_Gly680fs)	Novel
8	F	2/6 mo	ALT 150 AST 210 GGT 24 SBA 160 INR 1.05 TSB/DSB 4.5/3.8	Severe cholestasis, fibrosis stage 2/3 according to Batts and Ludwig classification (6 mo)	PFIC, liver cirrhosis	LTx (1 y 4mo)	*ABCB11*	c.185T>A, p.(Met62Lys)/c.1544A>C, p.(Asn515Thr)	Known (20232290)/Known (16641580)
9	M	2/6 mo	ALT 90 AST 106 GGT 11 SBA 507 INR 0.92 TSB/DSB 8.8/8.5	Severe cholestasis, fibrosis stage 2 according to Batts and Ludwig classification (6 mo)	PFIC, liver cirrhosis	LTx (3 y 6 mo)	*ABCB11*	c.1081C>T, p.(Gln361^*^)/c.1445A>G, p.(Asp482Gly)^*^	Novel/known (9806540)
10	M	6/6 mo	ALT 80 AST 100 GGT 20 SBA 100 INR 1.05 TSB/DSB 3.2/2.5	Moderate cholestasis, fibrosis stage 1/2 according to Batts and Ludwig classification (6 mo)	PFIC		No pathogenic molecular variants identified in NGS targeted gene panel		
11	M	11 mo/2 y	ALT 67 AST 49 GGT 24 SBA 24.6 INR 1.05 TSB/DSB 0.5/0.4	Fibrosis stage 3 according to Batts and Ludwig classification, minimal cholestasis (2 y)	PFIC		No pathogenic molecular variants identified in NGS targeted gene panel		
**GROUP 2B**									
12	M	1/6 mo	ALT 316 AST 1178 GGT 36 SBA 154 INR 1.83 TSB/DSB 20.85/16.0	Severe cholestasis, fibrosis stage 3/4 according to Batts and Ludwig classification, hepatitis gigantocellularis (6 mo)	Undeterminant cholestatic liver cirrhosis	LTx (8 mo)	*CYP27A1*	c.1184+1G>A, p.?/c.1184+1G>A, p.?	Known (9392430)
13	F	4/5 y	ALT 114 AST 59 GGT 155 SBA 3.5 INR 1.75 TSB/DSB 7.0/1.5	Severe cholestasis, fibrosis stage 2/3 according to Batts and Ludwig classification (5y)	Undeterminant cholestatic liver cirrhosis	LTx (12 y)	*ABCB4*	c.1745G>A, p.(Arg582Gln)/c.2149T>A, p.(Cys717Ser)	Known (22343912)/known (31115781)
14	M	1 mo	ALT 40 AST 90 GGT 480 SBA 415 INR 1.75 TSB/DSB 7.0/1.5	Minimal steatosis, severe cholestasis, fibrosis stage 3 according to Batts and Ludwig classification	Undeterminant cholestatic liver cirrhosis	LTx (6 mo)	*DGUOK*	c.1A>G, p.?/c.3G>A, p.?	Known (16908739)/known (16908739)
15	M	2/2 mo	ALT 244 AST 360 GGT 106 SBA 465 INR 1.33 TSB/DSB 8.2/7.3	Severe cholestasis, fibrosis stage 1 according to Batts and Ludwig classification, Hepatitis gigantocellularis (2 mo); Severe cholestasis, fibrosis stage 3 according to Batts and Ludwig classification, Hepatitis gigantocellularis (6 mo)	Undeterminant cholestatic liver cirrhosis	LTx (10 mo)	No pathogenic molecular variants identified in NGS targeted gene panel		
16	M	1/1 mo	ALT 88 AST 129 GGT 18 SBA n.a. INR 1.51 TSB/DSB 29.1/22.0	Severe cholestasis, fibrosis stage 1, Hepatitis gigantocellularis (1 mo); Fibrosis stage 3, hepatitis gigantocellularis (2 mo)	Undeterminant cholestatic liver cirrhosis	LTx (1 y 2 mo)	No pathogenic molecular variants identified in NGS targeted gene panel		
17	M	6 /7 mo	ALT 66 AST 97 GGT 958 SBA n.a. INR 1.14 TSB/DSB 0.73/0.4	Severe cholestasis, liver cirrhosis (7 mo)	Undeterminant cholestatic liver cirrhosis	LTx (5 y)	No pathogenic molecular variants identified in NGS targeted gene panel		

*ALT, alanine aminotransferase, n <60 U/l; AST, aspartate aminotransferase, n <84 U/l; GGTP n <200 U/l (till 3 months); TSB, total serum bilirubin, n <1.0 mg/dl; DSB, direct serum bilirubin, n <0.5 [mg/dl]; INR, internal normalized ratio, n = 0.8–1.2; n.a., not analyzed; mo, months; y, years; F, female; M, male; PMID, PubMed unique identifier*.

1 *The nomenclature of identified variants follows the Human Genome Variation Society guidelines (HGVS v 2.0, www.hgvs.org/mutnomen) and referral to the cDNA and protein sequences: NM_000443.3, NP_000434.1 for ABCB4; NM_003742.2, NP_003733.2 for ABCB11; NM_000784.3, NP_000775.1 for CYP27A1; NM_080916.2, NP_550438.1 for DGUOK and NM_004817.3, NP_004808.2 for TJP2 followed the Human Gene Mutation Database (HGMD, www.hgmd.cf.ac.uk). Boldface print, variants known in HGMD Professional 2019.4*.

2 *Other pathogenic molecular variants not identified*.

* *The recurrent mutation, identified on six alleles in five individuals; variant is also frequent in different population databases: gnomAD (0.0022% in general and 0.0047% in European Non-Finnish population) and in Polish population (0.2%)*.

## Discussion

The study presented a high clinical utility of panel-based NGS in the diagnostic approach of monogenic cholestatic liver diseases in pediatric hepatology and liver transplant center.

There have been published a few studies which have assessed a comprehensive molecular analysis approach based on high-throughput sequencing (panel-based NGS) for neonatal/infantile cholestasis (12–15). The diagnostic yield of panel-based NGS was reported to be various, mostly depending on the patients selection criteria. The diagnostic yield in our study was high because the patients were enrolled in a referral center with facilities of liver transplantation. The highest diagnosis rate was noted to identify the molecular background of cholestatic liver diseases presenting with a low-GGT PFIC phenotype. Similarly to the literature, the important value of applying NGS was reported in cases of an early onset cholestasis to rapidly identify the molecular background of disorders causing low-GGT PFIC (12–15).

Togawa et al. (12) performed the study on usefulness of NGS panel containing 18 genes in children with neonatal/infantile intrahepatic cholestasis. Out of 109 patients recruited, 31 (28%) were molecularly diagnosed. This low diagnostic yield could be related to the patients selection criteria, because besides the patients clinically diagnosed with known genetic cholestasis, there were included children with probable non-genetic causes such as prematurity, infections, metabolic, and endocrinological abnormalities. Other cause of low diagnostic yield could be using NGS panel with only 18 genes compared to the gene panel with 53 genes applied in our studies. The positive molecular diagnosis rate in the group clinically diagnosed with known genetic cholestasis syndromes such as Alagille syndrome, PFIC, neonatal intrahepatic cholestasis caused by citrin deficiency and Dubin–Johnson syndrome was 71% (22/31). This rate is comparable with our results regarding the group presenting with low-GGT PFIC phenotype.

In the study of Chen et al. (13), 102 patients presenting with various forms of cholestasis or idiopathic liver disease were recruited and subsequently underwent a molecular analysis by NGS panel containing 52 genes. The overall diagnostic yield of genetic diagnoses was 33 of 102 (32%) patients. A greater diagnostic yield was noted in 25 of 37 (68%) patients with suspected genetic diseases including PFIC (16/27, 59%), Wilson disease (5/5, 100%), or syndromic cholestasis (4/5, 80%). The latter results are comparable with ours highlighting the usefulness of panel-based NGS in diagnosing patients with genetic liver diseases in a proper clinical setting.

Nicastro et al. (14) have recently presented a diagnostic protocol addressed to all newborns and infants with persistent cholestasis. The stool color, GGT level, and liver biopsy pattern were the main determinants aiming to timely diagnose the extrahepatic causes of cholestasis and then, define the group of patients with suspected monogenic diseases. Overall, genetic testing allowed a final diagnosis in 29 of 48, with a 60% detection rate. Among the inherited disorders identified, the most frequent were Alagille syndrome and PFIC1–3. According to Nicastro et al. (14) we acknowledge that targeted NGS appears to be a very useful tool in diagnosis of monogenic cholestatic liver disorders in cases when extrahepatic causes have been primarily excluded.

The diagnostic approach of neonatal/infantile cholestasis is traditionally based on clinical, biochemical, imaging, and histopathological findings (7). The identificable causes of cholestasis have grown recently due to application of NGS technology (6). Molecular analysis is also helpful to expand the genotypic spectrum of diseases presenting with similar phenotypes (6). The pathogenic variants in TJP2 (encoding the TJP2 protein which is essential for tightness of cell junctions between the epithelial cells lining the bile ducts), NR1H4 (encoding the regulatory FXR of bile acids synthesis and enterohepatic flow) and *MYO5B* genes have been found recently in patients presenting with phenotype resembling BSEP deficiency (16–18). In our study we found two patients to be affected with TJP2 deficiency presenting with low-GGT PFIC phenotype.

There are also some diagnostic methods like urine bile acids analysis using liquid secondary ionization mass spectrometry, that are not routinely available worldwide yet (19). The introduction of targeted NGS allowed us to diagnose disorders which have not been recognized yet, like inborn errors of bile acids metabolism (CYP27A1 deficiency).

The molecular characterization constitutes the essential determinant for making decisions about the medical management, including liver transplantation (LTx). Currently, we know about the development of progressive fatty liver disease in some FIC1 deficient patients after Ltx (20, 21). LTx still remains controversial in the group of hepatic mitochondrial DNA depletion syndromes due to an extrahepatic involvement, possible neurological deterioration and unpredictable outcome (19, 22). However, in our center, like in the others worldwide, LTx is sometimes performed before molecular confirmation of the underlying disease. That is why, targeted NGS is essential to timely diagnose the underlying monogenic disease. In our study, NGS helped to identify one patient with mild form of DGUOK deficiency.

NGS sequencing is also very useful in diagnosis of ABCB4 deficiency due to its heterogeneous clinical presentation (23). Dröge et al. (24) in the cohort of 427 patients with suspected genetic cholestasis observed that the median age of symptoms onset in PFIC-3 patients was later than in patients with PFIC-1 or PFIC-2. Schatz et al. (25) have also described the national survey data on ABCB4 disease highlighting that the younger disease onset, the more severe phenotype, and patients with milder phenotypes could be not diagnosed before adulthood. In our study, we molecularly diagnosed two patients with ABCB4 deficiency—the first one with milder phenotype and symptom onset in adulthood and the second one with more severe phenotype requiring LTx early in life.

Although the presented information is not new, the study provided useful information documenting the diagnostic value of NGS panel in cholestatic liver disease, especially those with low-GGT cholestasis. One missense mutation p.Asp482Gly was the most frequent in our patients. Novelty was given by description of new pathogenetic variants for cholestatic liver diseases.

## Conclusions

1. Panel-based NGS appears to be a very useful tool in diagnosis of monogenic cholestatic liver disorders in cases when extrahepatic causes have been primarily excluded.
2. NGS presented the highest diagnosis rate to identify the molecular background of cholestatic liver diseases presenting with a low-GGT PFIC phenotype.

## Data Availability Statement

The raw data supporting the conclusions of this article will be made available by the authors, without undue reservation, to any qualified researcher.

## Ethics Statement

An informed and written consent was obtained from patients and their parents. Ethical approval of the study protocol was obtained from the Children's Memorial Health Institute Bioethical Committee, Warsaw, Poland.

## Author Contributions

Conception and design of study: PL and IJ. Analysis and/or interpretation of data: PL, EC, DJ, AP, MW, RP, JC-K, PS, JP, and IJ. Drafting the manuscript: PL, EC, and DJ. Revising the manuscript: JP and IJ.

## Conflict of Interest

The authors declare that the research was conducted in the absence of any commercial or financial relationships that could be construed as a potential conflict of interest.

## Abbreviations

BSEP: bile salt export pump
CYP27A1 deficiency: sterol 27-hydroxylase deficiency
DBS: dried blood spot
DGUOK deficiency: deoxyguanosine kinase deficiency
ESPGHAN: European Society for Pediatric Gastroenterology, Hepatology and Nutrition
GC/MS: gas chromatography-mass spectrometry
GGT: gamma-glutamyl transferase
IEF: isoelectric focusing
LTx: liver transplantation
MS/MS: tandem mass spectrometry
NGS: next-generation sequencing
PEBD: partial external biliary diversion
PFIC: progressive familial intrahepatic cholestasis
TJP-2: tight junction protein 2
WES: whole-exome sequencing.
